# Change Analysis in Structural Laser Scanning Point Clouds: The Baseline Method

**DOI:** 10.3390/s17010026

**Published:** 2016-12-24

**Authors:** Yueqian Shen, Roderik Lindenbergh, Jinhu Wang

**Affiliations:** 1 School of Earth Science and Engineering, Hohai University, No. 1, Xikang Road, Nanjing 210098, China; 2 Department of Geoscience and Remote Sensing, Delft University of Technology, Stevinweg 1, 2628 CN Delft, The Netherlands; r.c.lindenbergh@tudelft.nl (R.L.); jinhu.wang@tudelft.nl (J.W.); 3 Key Laboratory of quantitative Remote Sensing Information Technology, Academy of Opto-Electronics, Chinese Academy of Sciences, No. 9, Deng Zhuang South Road, Haidian District, Beijing 100094, China

**Keywords:** terrestrial laser scanning, change detection, masonry buildings, baselines, structural analysis

## Abstract

A method is introduced for detecting changes from point clouds that avoids registration. For many applications, changes are detected between two scans of the same scene obtained at different times. Traditionally, these scans are aligned to a common coordinate system having the disadvantage that this registration step introduces additional errors. In addition, registration requires stable targets or features. To avoid these issues, we propose a change detection method based on so-called baselines. Baselines connect feature points within one scan. To analyze changes, baselines connecting corresponding points in two scans are compared. As feature points either targets or virtual points corresponding to some reconstructable feature in the scene are used. The new method is implemented on two scans sampling a masonry laboratory building before and after seismic testing, that resulted in damages in the order of several centimeters. The centres of the bricks of the laboratory building are automatically extracted to serve as virtual points. Baselines connecting virtual points and/or target points are extracted and compared with respect to a suitable structural coordinate system. Changes detected from the baseline analysis are compared to a traditional cloud to cloud change analysis demonstrating the potential of the new method for structural analysis.

## 1. Introduction

Terrestrial Light Detection and Ranging (Lidar) generates 3D coordinates of an object point by measuring the horizontal and vertical angle and the distance between the scanner’s centre and the object point. Due to its ability to provide dense, fast and accurate measurements, the use of terrestrial Lidar for various surveying applications such as deformation monitoring and damage detection has increased rapidly [1]. Terrestrial Lidar is used in civil engineering applications in structural monitoring of tunnels [2], road modelling [3], sign inventory [4], evaluating deformations and/or geometric changes [5], road identification [6], drift detection [7], natural hazards and structural analyses in cultural heritage [8,9]. The application of terrestrial Lidar data for accurately measuring deflection of loaded beams is discussed by Park et al. [10]. 

The density of points on the object’s surface can be predefined by the user and is limited by the minimum angle increment of the system. Depending on the distance from the scanner and the amount of scans, a very high point density can be achieved. In addition, the reflectance of the surface may be measured by recording the intensity of the reflected laser beam. 

Combined with high quality static GNSS positioning and precise tachometry, terrestrial Lidar was used for high precision monitoring of deformations during longer time intervals [11]. A terrestrial Lidar-based vehicle detection approach was proposed by using the so-called Probability Hypothesis Density [12]. Automatic processing of point clouds was carried out to so-called build geometric models suitable for structural analysis purposes [13]. A method used the triangulation, reflectance and RGB triplets to obtain a proximity-based segmentation [14]. In recent decades, the advanced analysis of masonry structures in large point clouds also received considerable attention [15]. A novel segmentation algorithm was proposed to enable the automatic segmentation of masonry blocks from 3D point clouds acquired by terrestrial Lidar technology [16]. Despite all the improvements, the challenges in the seismic assessment of these structures remains rather difficult [17]. What’s more, these type of structures are quite large due to the numerous variations of masonry, the large scatter of in situ material properties, and the impossibility of reducing it all in a specimen.

Change detection on point clouds is a rather new technique considering that it has been mainly used by professional surveyors until now [18]. Comparison of the surface geometry in different epochs is often performed by reconstructing the surface models in each epoch [19,20]. By comparing the resulting surface models, useful change information is obtained. Combined with conventional surveying devices such as total station and GPS, georeferenced topographic data is acquired by terrestrial Lidar for further scene comparison [21]. Subtraction of a resampled data set was used to detect changes on a hydropower station [22]. One common way to perform change detection is to compare the 3D coordinates of corresponding points from two epochs. Before the comparison, two point clouds should be aligned in the same coordinate system which is called registration. Therefore, registration is a crucial step in change detection applications. Point clouds can be registered by iteratively decreasing the distances to arbitrary closest points in overlapping areas, which is the basis of Iterative Closest Point (ICP)-like algorithms [23,24], or by matching explicitly derived features points [25,26,27,28]. However, even minor misalignments of two epochs may lead to erroneous results when detecting changes. Another common technique, to perform change detection on a point cloud, which also requires registration in advance, is to compute its distance to a 3D reference model. This can be done either directly or by creating an intermediate model on top of the points. The reference model can either be theoretical or also created from real data. Cloud-to cloud and cloud-to-mesh distances have been very well studied based on above principles. The academic software, “Metro” and “Mesh”, allows one to compare the difference between a pair of surfaces by adopting a surface sampling approach [29]. An efficient method was proposed to estimate the distance between discrete 3D surfaces represented by triangular 3D meshes which was based on an approximation of the Hausdorff distance [30]. Several simple cloud-to-cloud comparison techniques based on a specific octree structure were presented and implemented [31]. Although the feasibility and effectiveness of the cloud-to-cloud and cloud-to-mesh distances has been demonstrated in previous work, the absolute value of displacement can’t be estimated.

To overcome these issues we propose instead to compare corresponding baselines extracted independently in each of the two scans. Here a baseline is defined as a 3D line segment connecting two points in one scan. Two baselines from different scans are corresponding if they connect the same features. Feature identification and extraction have drawn many scholars’ attention in recent years. A laser-based approach for door and handle identification was proposed and implemented [32]. Euclidean was applied for tabletop object detection which was efficient and enables real-time plane segmentation [33]. The Fast Point Feature Histograms was used for labelling 3D points with different geometric surface primitives [34]. As features we propose to use two type of points. First, target points identified by spherical or planar targets as placed by the surveyor in the scene, and, second so-called virtual points, which give the 3D location of a feature that is well-reconstructable from the 3D scan data. What virtual points are suitable depends on the particular scene that is considered. 

This proposed methodology is illustrated to detect changes on a masonry building during seismic testing, mainly for monitoring purposes but also to be able to provide change information for further structural analysis. In this case, as in most cases, target points are easily identified in the scan data. The extraction of virtual points requires more work. In this case we choose as virtual points the centres of bricks. In the building scan, one wall that is expected to be stable is available, while another wall is actually cracked during the seismic testing.

The extraction of virtual points starts with separation of mortar and bricks in the scan data using k-means clustering of the TLS intensity attribute. Next, point clouds are segmented and 3D virtual point locations are estimated at the brick centres. Given both the resulting target points and virtual points, baselines are constructed. Finally, by comparing corresponding baselines from the two epochs, changes in X, Y and Z direction of a suitable structural coordinate system are extracted.

As an evaluation of the proposed approach, results are compared to results from traditional methods. That is, for a traditional approach, scan data is first registered in a common coordinate system. Next, virtual point changes and cloud-to-cloud distances between the aligned point clouds are estimated. As a first benefit of the proposed method we were able to identify a target that was apparently moved by an external agent early in the reconstruction. A detailed analysis of the change results will be presented in the results section. To summarize, the proposed method is a new, alternative approach for change detection that eliminates an often unnecessary registration step and its associated errors.

## 2. Materials and Methods

**Figure 1:**
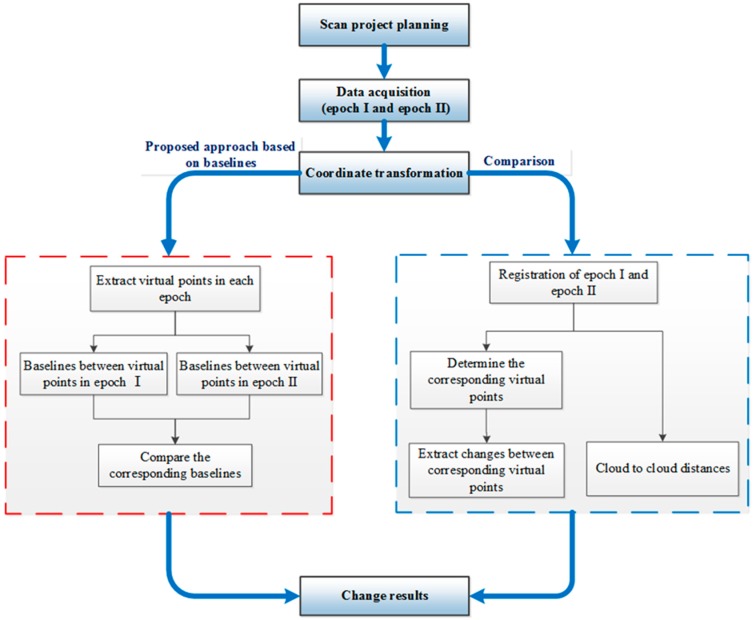
Change detection workflow.

In order to detect changes by baselines, a workflow is introduced in Figure 1. The left box summarizes the new baseline method while the right box shows the comparison approaches. 

### 2.1. Seismic Experiment Description and Scan Data Acquisition

A seismic experiment has been carried out on a masonry house in the Stevin lab of TU Delft. The purpose of the experiment was to explore how resilient houses are against earthquakes. The house was built of bricklayers with mortar and calcium silicate. The experiment was performed from 3 December 2015 to 16 December 2015. During the experiment the house was shaken back and forth repeatedly and repeated scanning was carried out.

A C10 Scan Station scanner (Leica, Heerbrugg, Switzerland) was used for this experiment. The Leica C10 Scan Station scanner is a time-of flight scanner with an effective range of 300 m at 90% reflectively. Specifications indicate that the accuracy of a single measurement is 6 mm (one sigma) in position and 4 mm (one sigma) in depth at ranges up to 50 m [35].

**Figure 2:**
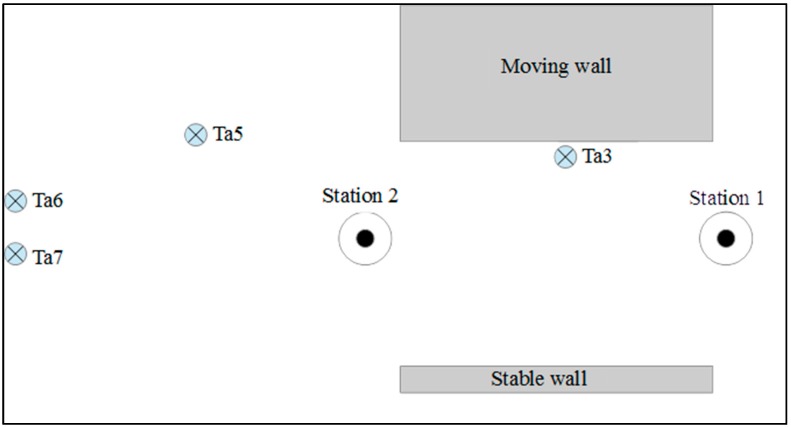
A sketch showing the position of the test building (moving wall), a stable wall and scan positions.

The location of the scan positions should be planned such that the number of scans is minimized while avoiding occlusions in order to ensure the full coverage of the whole monitoring area [36]. Actually, one station is enough to cover the façade of the moving masonry house in this project. However, we planned two stations, one on the left side of the house and the other on the right side, to get additional information about the changes. The positions of the moving wall, stable wall, the scanner and the targets are shown in a top view of the experiment scene, see Figure 2. The left side of the scene contains a big door and lacks stable locations for positioning further targets. But, as only the comparison methods need registration, the used target distribution has no effect on the results of the proposed method.

**Figure 3:**
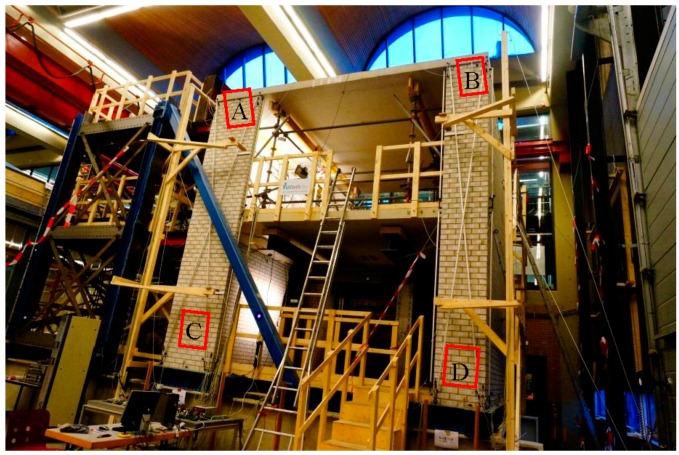
Station view of the seismic testing house.

Figure 3 is a picture of the moving wall taken from the perspective of Station 1. Considering the structure of the wall, areas of interest should cover the wall in a representative way. Therefore, patches A, B, C and D at the top-left, top-right, bottom-left and bottom-right of the moving wall respectively, are chosen as a source for virtual points, see Figure 3. Here we should demonstrate that if we choose the whole wall which contains too much outliers as well as the crakes and it will definitely complicate the pre-processing. Moreover, four patches are enough to show the structural change of the wall. Actually, our paper aim at demonstrating the proposed baseline method, so we only selected the above four patches.

Two scans were made from each scan position. The first scan was at a minimal resolution, which correspond to 0.2 m in horizontal and vertical spacing when the range is 100 m [37], with an entire field of vision (360 degree with respect to Z-axis and 270 degree for vertical amplitude) which is used for obtaining a general frame of the whole scanner field of view, where smaller areas can be selected for subsequent scans. The second high-resolution scan, which corresponds to 0.05 m in horizontal and vertical spacing at a range of 100 m [37], was of an area including the moving wall, stable wall and targets. Due to the different ranges to the scanner, the horizontal and vertical spacing at patch A, patch B, patch C and patch D is 0.0055 m, 0.0035 m, 0.0047 m and 0.0028 mm, respectively.

**Figure 4:**
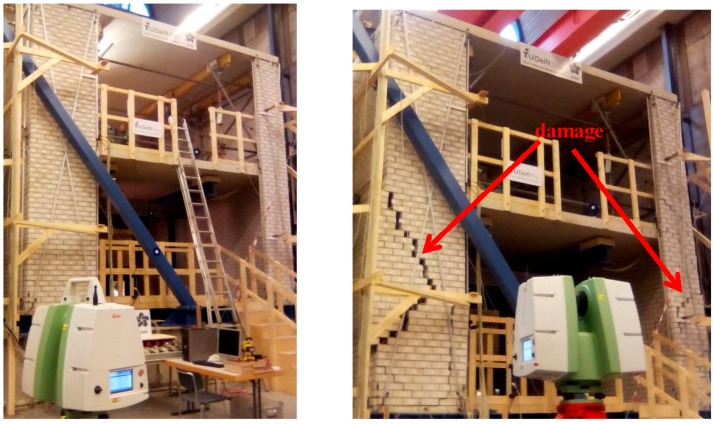
On the **left**: the masonry wall before the seismic testing; on the **right**: the damaged masonry wall after the seismic testing.

Two epochs of TLS data (on 3 December 2015 and 16 December 2015) were obtained before and after the seismic testing, see Figure 4, in which the induced damage is apparent. Each epoch was scanned from almost the same place. Together with the 3D coordinates of the points in the monitoring surface, the coordinates of the reference targets were also obtained. They are distributed away from the moving wall so that there are at least four common targets between scans of different epochs, compare Figure 2. 

### 2.2. Change Detection Based on Baselines

#### 2.2.1. Coordinate Transformation

**Figure 5:**
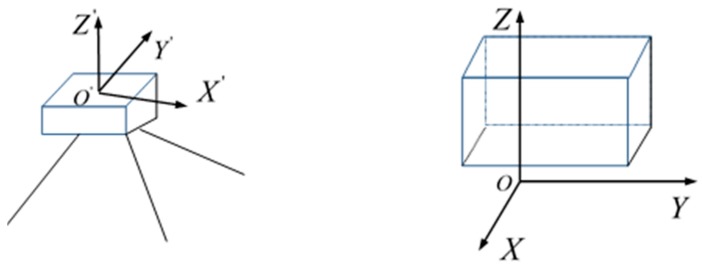
TLS (**left**) and structural (**right**) coordinate system.

In order to obtain more intuitive change information, a structural coordinate system is designed. The initial coordinates, $X^{\prime }$, $Y^{\prime }$ and $Z^{\prime }$ values obtained by the TLS, are determined by the location of the TLS (i.e., distance of TLS to the object and its orientation angle), see the left figure in Figure 5. Displacement in the $X^{\prime }$, $Y^{\prime }$ and $Z^{\prime }$ direction does not correspond to the expected changes of the structure (i.e., along or perpendicular to the building walls) which should be measured in the X, Y and Z direction of the structure. Therefore, we define a structural coordinate system as follows, see the right figure in Figure 5. Here, the Y and Z axes are parallel to the building walls. The Z axis is pointing upwards and the X axis is pointing out the building.

The 3D transformation from the TLS coordinate system to the structural coordinate system consists of six parameters, three translations (i.e., three parameters in three orthogonal directions) and three rotational parameters. The translation moves the origin $O^{\prime }\left({x_{0}}^{\prime },{y_{0}}^{\prime },{z_{0}}^{\prime }\right)$ in the TLS coordinate system to the origin $O\left(x_{0},y_{0},z_{0}\right)$ in the structural coordinate system, see Figure 5.

The testing building is part of a larger scene. The structural coordinate system is established on the stable place such as laboratory ground, ceiling surface, the experimental steel frame, fixed wall, and et al. In our processing, we select the fixed wall, which is parallel to the testing wall surface, to set up the structural coordinate system, compare Figure 2. The procedure is as follows:

1. Select the point cloud of the fixed wall.
2. Fit a plane to the point cloud using Principle Component Analysis (PCA).
  The PCA method is used to estimate the point clouds normal vector in our work [38,39,40].
3. Project the origin of the TLS coordinate system to the plane and calculate the translational parameter.
  The plane equation of the fixed wall is obtained by the last step. Afterwards, the origin $O^{\prime }$ of the TLS coordinate system is projected to the fixed wall, being the origin O of the structural coordinate system. Assuming that the plane equation of the fixed wall and the origin of TLS coordinate system are expressed as:
  (1) $$\begin{array}{l} Ax+By+Cz+D=0\\ O^{\prime }=(0,\;\;0,\;\;0) \end{array}$$
  The origin of the structural coordinate system is computed as:
  (2) $$O\left(\frac{-AD}{A^{2}+B^{2}+C^{2}},\;\frac{-BD}{A^{2}+B^{2}+C^{2}},\;\;\;\frac{-CD}{A^{2}+B^{2}+C^{2}}\right)$$
4. Calculate the rotational moves and transform the coordinates.
  The station is always level during scanning. Therefore, the structural coordinate system is established by rotating in the $X^{\prime }O^{\prime }Y^{\prime }$ plane. The normal vector $\vec{n}$ of the stable wall is regarded as the positive X axis. The rotation angle α is the angle between the normal vector $\vec{n}$ and the $X^{\prime }$ direction ${\vec{n}}_{X^{\prime }}=[1,0,0]$. Therefore, the 3D coordinate transformation is defined by:
  (3) $$\left[\begin{array}{c} X\\ Y\\ Z \end{array}\right]=\left[\begin{array}{ccc} \cos\alpha & -\sin\alpha & 0\\ \sin\alpha & \cos\alpha & 0\\ 0 & 0 & 1 \end{array}\right]\left[\begin{array}{c} X^{\prime }\\ Y^{\prime }\\ Z^{\prime } \end{array}\right]+\left[\begin{array}{c} \Delta X\\ \Delta Y\\ \Delta Z \end{array}\right]$$
  Here, α represents the rotation angle between $X^{\prime }O^{\prime }Y^{\prime }$ and XOY; ${\left[\Delta X\;\;\;\;\;\Delta Y\;\;\;\;\;\Delta Z\right]}^{\prime }$ denotes the translation vector, and in this paper, ${\left[\Delta X\;\;\;\;\;\Delta Y\;\;\;\;\;\Delta Z\right]}^{\prime }={\left(\frac{AD}{A^{2}+B^{2}+C^{2}},\;\frac{BD}{A^{2}+B^{2}+C^{2}},\;\;\;\frac{CD}{A^{2}+B^{2}+C^{2}}\right)}^{\prime }$.

#### 2.2.2. Automatic Virtual Point Extraction

As introduced above, a baseline is defined by connecting two feature points, thus it is necessary to select and extract obvious feature points (e.g., brick centres, door knobs, corner of the wall) from different areas, including moving areas, stable area and targets.

**Figure 6:**
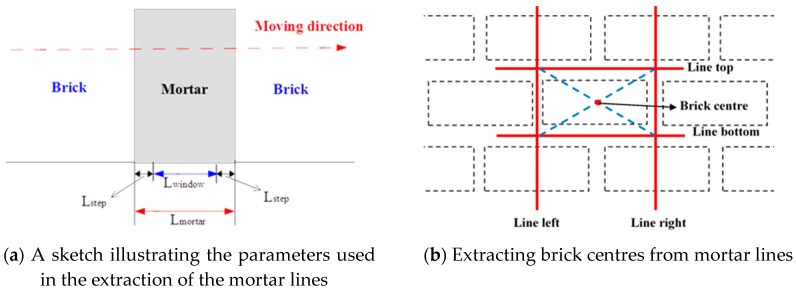
Automatic virtual point extraction.

In this research, we choose brick centres as our virtual points. To implement the proposed method, bricks without damage are selected and three assumptions are made about the bricks after the seismic test: (1) a brick is always rectangular; (2) no crack emerges; (3) brick movement was translational, not rotational. These assumptions are easily met in this case, but particular assumptions are case study dependent. In this study, each brick is sampled by several random points so we have to reconstruct corresponding points in corresponding bricks. To do so we perform three steps to be discussed in detail (1) separation of mortar and bricks; (2) construction of a histogram of mortar points; (3) estimating brick centres. These steps will enable us to extract 3D brick centres from the brick point clouds for detecting changes:

1. Separation of Mortar and Bricks Using K-means Clustering
  Laser scanners not only provide information about the geometric position of a surface, but also information about the portion of energy reflected by the surface, which depends on its reflectance characteristic. The backscatter generated after collision of the laser beam with the object surface is recorded by most terrestrial Lidar instruments as a function of time [41]. In our work, the mortar and the brick are composed of different materials, so the signal intensity attribute is considered a possibility to segment bricks from mortar using k-means clustering, a commonly used data clustering technique for performing unsupervised tasks. It is used to cluster n objects of the input dataset into k homogeneous partitions, k<n [42,43]. In our study, the wall is composed of brick and mortar so we use this technique in a classic way with k=2 to separate brick points from mortar points based on their intensity.
2. Histogram of Y and Z Axes Using Mortar Points
  The brick point clouds are obtained by the above steps but with no accurate boundaries between different bricks. The wall is coursed masonry which means the masonry and the mortar are almost level to the wall surface. In addition, the 3D coordinates of the points have been transformed to the structural coordinate system as introduced in Section 2.2.1, which indicates that the X axis is perpendicular to the plane of the masonry wall. Therefore, a histogram along the Y and Z axis is used to estimate boundary lines between different bricks. The possible horizontal and vertical boundary lines of the bricks are determined by the peaks of the histogram along the Y and Z axis using the mortar points extracted in step 1. The procedure of determining the lines is as follows:
  As shown in Figure 6a, a window width $L_{window}$ and a step width $L_{step}$ is defined along the Y or the Z axis which satisfies the equation on the width of the mortar $L_{mortar}$ between two bricks as follows:
  (4) $$L_{mortar}\approx L_{window}+2L_{step}$$
  In our work, the number of window positions along Y-direction and Z direction is given by:
  (5) $$n_{y}=\left[\frac{y_{\max}-y_{\min}-L_{window}}{L_{step}}\right]+1,\;\;\;\;\;n_{z}=\left[\frac{z_{\max}-z_{\min}-L_{window}}{L_{step}}\right]+1$$
  In which the sign [ ] means that the value is rounded up toward the nearest integer.
  The number of points $n_{yi}\left(i=1,\;2,\cdots ,n_{y}\right)/n_{zi}\left(i=1,\;2,\cdots ,n_{z}\right)$ in each window is computed and the (x,y,z) coordinates of the points are stored.
  The “line density” along the Y- or Z-direction is expressed as:
  (6) $$\begin{array}{l} Density\_y=\left(n_{y(i-1)}+n_{yi}+n_{y(i+1)}\right)/\left(3L_{window}\right)\;\left(i=2,3,\cdots ,(n_{y}-1)\right)\\ Density\_z=\left(n_{z(i-1)}+n_{zi}+n_{z(i+1)}\right)/\left(3L_{window}\right)\;\left(i=2,3,\cdots ,(n_{z}-1)\right) \end{array}$$
  For each window, the line density gradient along the Y-direction or Z-direction is calculated by:
  (7) $$\begin{array}{l} Grad\left(i,1\right)=Density\_y(i)-Density\_y(i-1)\\ Grad\left(i,2\right)=Density\_y(i+1)-Density\_y(i) \end{array}$$
  When Grad(i,1)>0 and Grad(i,2)<0, the window of the mortar is determined. For the analyzed directions, it always has a higher density when the mortar is perpendicular to this direction. Therefore, a density threshold is considered when the mortar window is estimated. In our work, the average number in each window width is taken as the threshold. That means that along the Y-direction and Z-direction, the thresholds are $\varepsilon_{y}=\frac{n_{total}}{n_{y}}$ and $\varepsilon_{z}=\frac{n_{total}}{n_{z}}$, in which $n_{total}$ represents the number of points belonging to this patch.
3. Estimating the Brick Centre Position
  The centre line position is estimated by calculating the average of the points in the mortar window as shown in Figure 6b. Four lines surrounding one brick are estimated by above procedure. Given the equations of the four lines, four intersection points are estimated. Next, the 3D coordinates of the brick centre are calculated by averaging the four intersection points. Finally, the extracted virtual points are sorted to facilitate automatic identification of virtual points from the same brick in different epochs. The regular configuration of the bricks in the masonry wall facilitates the automatic handling of the brick centre locations.

#### 2.2.3. Baseline Establishment

In our work, a baseline is defined as a 3D line segment connecting two points in one scan. The points represent features such as points identified by spherical or planar targets placed by a surveyor in the scene and so-called virtual points extracted from the 3D scan data. We propose to use two type of baselines for change detection which are established by connecting points that are expected to indicate structural change of the moving wall. First, points subject to change are connected to points that are expected to be stable, and, second points subject to change are connected to each other.

**Figure 7:**
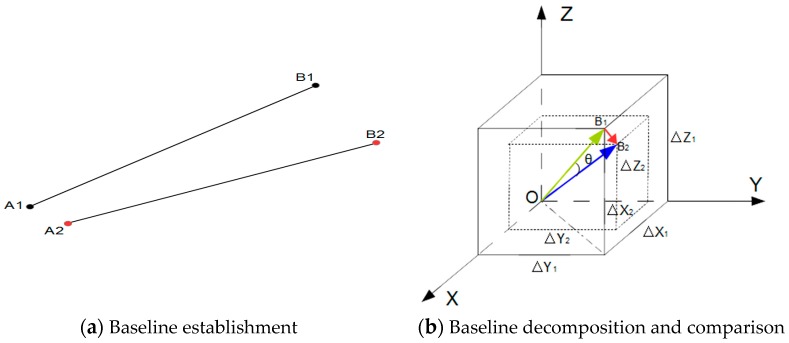
Baseline establishment, decomposition and comparison.

By connecting two different points, a baseline is established. In Figure 7a, assuming that $A_{1}$ and $B_{1}$ represent the points in epoch I while $A_{2}$ and $B_{2}$ are the corresponding points in epoch II, the baselines $A_{1}B_{1}$ and $A_{2}B_{2}$ are the corresponding baselines within one scan in different epochs. This step is performed in an automated way, which is possible due to the previous sorting of the extracted virtual points. 

#### 2.2.4. Baseline Decomposing and Comparison

For each pair of corresponding baselines from two epochs, a displacement vector is computed. Next, the length change and the change projected to the X, Y, Z direction, of the baselines are calculated. Actually, the start of every 3D vector can be translated to the origin O of the coordinate system. Therefore, corresponding baseline vectors from two epochs have the same beginning (i.e., O represents $A_{1}$ and $A_{2}$ in epochs I and II). A graphical illustration is shown in Figure 7b: $\vec{OB_{1}}$ and $\vec{OB_{2}}$ represent corresponding baseline vectors in epochs I and II, thus, $\vec{B_{1}B_{2}}$ is the displacement vector while θ is the rotation angle between the two baseline vectors;$\Delta X_{1}$, $\Delta Y_{1}$ and $\Delta Z_{1}$ are the coordinates of the baseline vector in epoch I while $\Delta X_{2}$, $\Delta Y_{2}$ and $\Delta Z_{2}$ are the coordinates of the baseline vector in epoch II. Actually, the baseline change includes the length change and the rotation change. The rotation change is finally transformed to the three axes direction of the structural coordinate system. Therefore, the changes could be estimated as long as determining the direction of the axes which is significantly different from registration.

### 2.3. Traditional Change Detection Methods

In order to validate the effectiveness of the proposed approach, the baseline method is compared to two traditional methods: direct virtual points comparison and cloud-to-cloud distances.

#### 2.3.1. Registration of Two Epochs

In general, the locations of scanning stations are not the same in different epochs, so the coordinates of identical points sampled in consecutive epochs are not expected to be equal either. In traditional change detection methods, two point clouds from two epochs are expected to be in a common coordinate system. Therefore, registration of two epochs is a vital pre-processing step required before detecting changes except for those monitoring applications where scan locations as well as targets positions are fixed by forced centering. Such setup guarantees that the point cloud acquired at each epoch is referred to the same coordinate system and therefore avoids registration. However, it is often difficult in practice to fix both scan locations and targets by forced centering as this requires additional preparation. Registration aligns and combines multiple data into a single set of range data. Registration may introduce small misalignment errors. Even minor misalignments of two epochs may lead to erroneous results during detecting changes. In this case study, the registration is based on control points.

The Leica Cyclone software provides an automatic registration method, with targets made by special materials. In this seismic testing experiment, we set four plane targets in fixed areas as control/tie points for registration. 

#### 2.3.2. Virtual Points Extraction and Comparison

Comparing corresponding points from different epochs [44] is a common way to obtain change information. Here we consider two ways, to extract virtual points automatically by the procedure discussed in Section 2.2.2 or manually. After determining virtual points and their correspondence from two epochs, the changes in X, Y and Z direction are computed.

#### 2.3.3. Cloud-To-Cloud Distances

**Figure 8:**
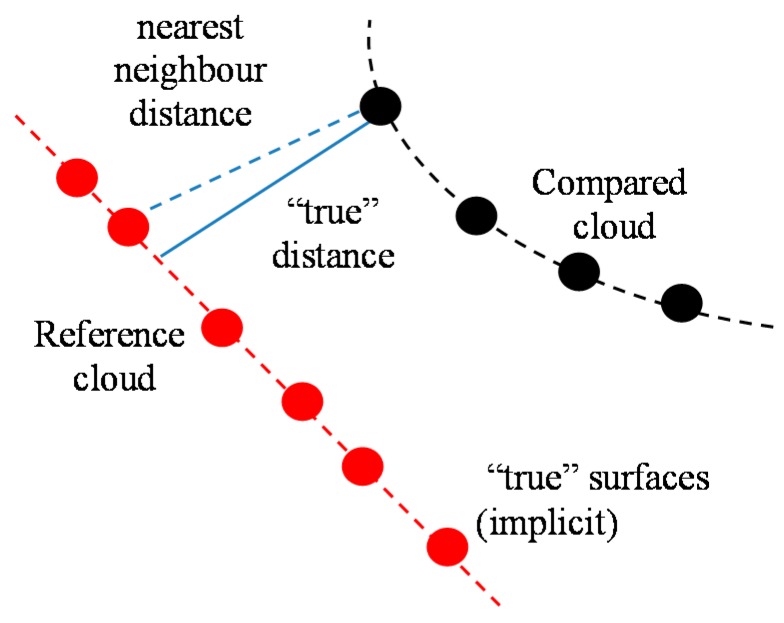
True global model for cloud-to-cloud distances.

It’s generally not so easy to get a clean and proper global model of a surface. Therefore, the idea of cloud to cloud distance is proposed which is the classical way to detect changes. The principle of nearest neighbour distance is used to compute distances between two points: for each point in the compared cloud, the nearest point in the reference cloud is searched and their Euclidean distance is computed [45], see Figure 8.

**Figure 9:**
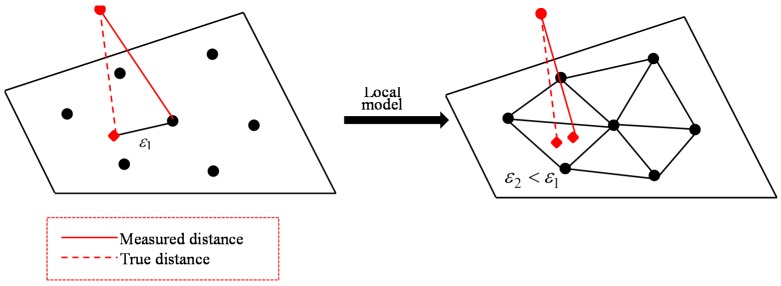
Local surface model for cloud-to-cloud distances.

Another immediate way, getting a better approximation of the true distance to the reference surface, is to get a local surface model. When the nearest point in the reference cloud is determined, the idea is first to locally model the surface of the reference cloud by fitting a mathematical primitive on the ‘nearest’ point and several of its neighbours, see Figure 9. The distance to this local model is finally reported. Common ways to locally model a surface are by triangles, compare Figure 9, by planes or by otherwise smooth patches. The effectiveness of this method is statistically more or less dependent on the cloud sampling and on how appropriate the local surface approximation is. 

To profit from observation redundancy, least square fitting is used to fit a plane or a higher order model to the points of the reference cloud.

## 3. Results

The results of brick centre extraction are presented below. Next, the resulting virtual points are used to establish baselines. Then, differences in corresponding baselines are presented as change detection results and compared to direct virtual point comparison and cloud-to-cloud distances results.

### 3.1. Structural Coordinate System Establishment and Coordinate Transformation

In order to facilitate interpretation of the results, we introduce a so-called structural coordinate system. Within this coordinate system, obtained 3D change vectors are roughly decomposed into wall-parallel and wall-perpendicular change. As shown in Figure 2, the points of the stable wall were selected to establish a structural coordinate system according to the principle introduced in Section 2.2.1. The plane equations in epoch I and epoch II are as follows:

Epoch I:0.9348x+0.3552y+0.0015z−8.8534=0;

Epoch II:0.9974x−0.0727y−0.0002z−8.8518=0;

The translation vector and the rotation angle are calculated between TLS and reference plane. Because the scanner is always level, the Z axis is the same as the $Z^{\prime }$ axis, and the normal vector of the stable wall is regarded as the positive the $X^{\prime }$ axis. Therefore, the 3D coordinate transformation can be defined by:

$$\text{Epoch I}:\left[\begin{array}{l} X^{\prime }\\ Y^{\prime }\\ Z^{\prime } \end{array}\right]=\left[\begin{array}{rrr} 0.9348 & -0.3552 & 0\\ 0.3552 & 0.0094 & 0\\ 0.0000 & 0.0000 & 1 \end{array}\right]\left[\begin{array}{l} X\\ Y\\ Z \end{array}\right]+\left[\begin{array}{l} -8.2760\\ -3.1451\\ -0.0132 \end{array}\right];$$

$$\text{Epoch II}:\left[\begin{array}{l} X^{\prime }\\ Y^{\prime }\\ Z^{\prime } \end{array}\right]=\left[\begin{array}{rrr} 0.9974 & 0.0727 & 0\\ -0.0727 & 0.0094 & 0\\ 0.0000 & 0.0000 & 1 \end{array}\right]\left[\begin{array}{l} X\\ Y\\ Z \end{array}\right]+\left[\begin{array}{r} -8.8284\\ 0.6432\\ 0.0020 \end{array}\right];$$

Finally, point clouds from both epochs are transformed from the TLS coordinate system to the structural coordinate system, respectively.

### 3.2. Virtual Point Extraction Results

#### 3.2.1. Automatic Method

The results of extracting virtual points automatically are shown in this section. Here, we take patch A as example to illustrate the method. 

**Figure 10:**
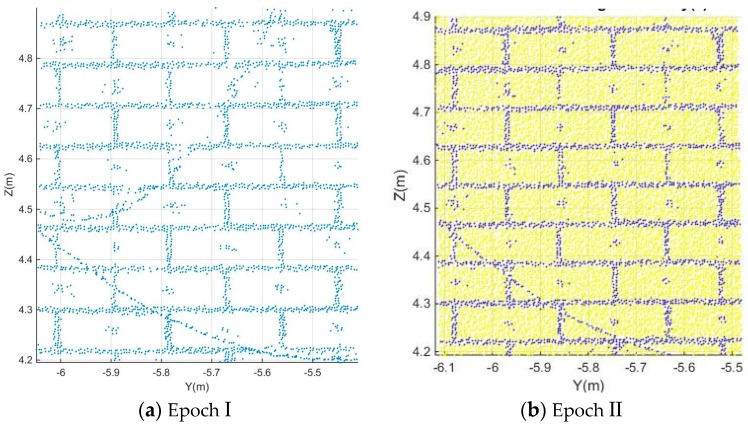
Results of separating brick points from notably mortar points in patch A using k-means clustering, with k = 2. Blue points correspond to mortar points, point reflecting from small photogrammetric targets attached to the brick centres, or to some wires in front of the wall. The yellow points in general belong to brick surfaces.

1. First, the brick and mortar points are separated by k-means clustering based on their intensity. Figure 10a shows the extracted mortar points in epoch I while Figure 10b shows both brick and mortar points in different colors after separation in epoch II. Other researchers, using photogrammetry, pasted small black targets at the centre of each brick. As a result, brick centre points appear to have the same color as mortar points. However, we estimated the brick centre locations from lines passing through mortar point. Therefore, the appearance of the brick centres will not affect our results. 

2. Resulting mortar points are projected to the Z and Y axis respectively. Through in situ measuring by steel ruler, the width of the mortar is estimated at $L_{mortar}=0.012m$. Therefore, the window width and step width are defined as $L_{window}=0.008m$ and $L_{step}=0.002m$ based on the equation $L_{mortar}\approx L_{window}+2L_{step}$ introduced in Section 2.2.4. Afterwards, the brick boundary lines are estimated by the principle introduced in Section 2.2.4.

**Figure 11:**
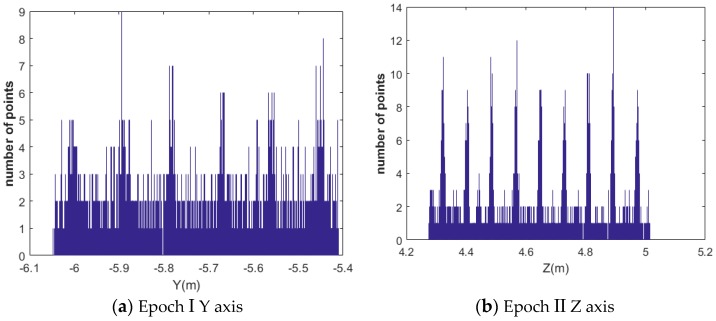
Histogram of mortar point frequencies along the Z and the Y axes.

The histograms of the number of points along Z and Y axes are shown in Figure 11. Figure 11a presents the histogram of the number of points along the Y axis in epoch I while Figure 11b shows the histogram of the number of points along the Z axis in epoch II.

**Figure 12:**
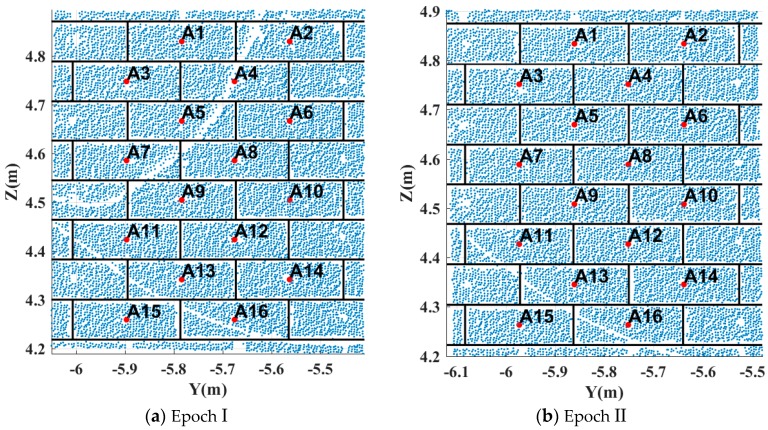
Virtual point extraction results (patch A). The red dots are the final virtual points.

3. Finally, the 3D coordinates of all brick centres are estimated by implementing the method proposed in Section 2.2.2. Patch A contains 16 bricks; Patch B contains three bricks; Patch C contains 15 bricks; Patch D contains six bricks. Figure 12 shows the extraction results of patch A in epochs I and II.

#### 3.2.2. Comparison to Manual Virtual Point Extraction

The automatic extraction is evaluated against manual picking the centre points of each brick. A human operator imported the two aligned point clouds to the “Cloudcompare” software and picked points representing the brick centres. By considering the study cases patch A, B, C and D, which contain a total of 40 bricks, in the moving wall, the average difference between manually picking and automatic extracting are [−0.23,1.09,−1.27] mm in the X, Y and Z direction respectively. The standard deviation along the X, Y and Z direction is 0.92, 2.48 and 1.44 mm, respectively. This gives an indication for the effectiveness and feasibility of the proposed automatic extraction method.

### 3.3. Baseline Establishment and Decomposing

**Figure 13:**
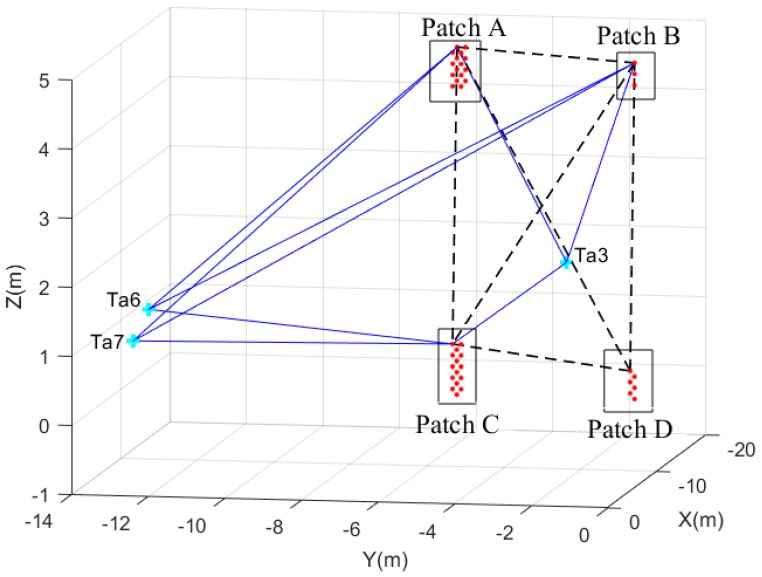
Baselines from virtual points to target points are indicated by continuous lines, while baselines connecting virtual points are shown as dashed lines.

To establish the baselines, we first select the four patches A, B, C and D. In addition, target points are identified. Through either linking different virtual brick centre points as extracted from the patches, or linking virtual brick centre points to target points, baselines are established. Figure 13 contains 18 such baselines. The 12 baselines shown as continuous lines, link brick centres in patch A, B, C and D to the three targets Ta3, Ta6 and Ta7. The six baselines shown as dashed lines, link brick centres to other brick centres.

By comparing corresponding baselines in two epochs, change vectors are estimated. Then, the change vectors are projected to the X, Y and Z axes within the structural coordinate system.

### 3.4. Baseline Changes

#### 3.4.1. Baseline Changes between Patches and Targets

**Figure 14:**
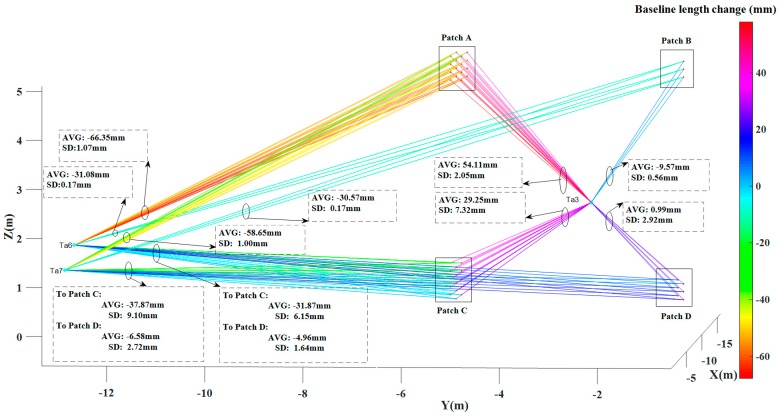
Baseline length changes (from target points to virtual brick centre points). The quantitative results are summarized in Table 1.

Figure 14 shows the length change for a total of 120 pairs of corresponding baselines between target points and virtual brick centre points, in which “Avg” represents the mean baseline length change and “SD” represents the standard deviation of the baseline length change. Here mean and standard deviation are taken from all pairs of corresponding baselines connecting the same patches and/or targets. The maximum length change, from target “Ta6” to the virtual brick centre point “A16” in patch A, is −67.79 mm in which the minus sign indicates that the length of baseline “Ta6-A16” in epoch II becomes shorter compared to epoch I. All the baselines from “Ta6” and “Ta7” to virtual brick centre points became shorter. This indicates that the moving wall as a whole tilted to the left during the experiment. The baselines from “Ta3” to virtual brick centre points in patch A and patch C become longer, while baselines from “Ta3” to virtual brick centre points in patch B and patch D became shorter. This contrasting pattern could match the previous pattern of the moving building tilting to the left: patch A and patch C move away from “Ta3”, while patches B and D move towards “Ta3”.

For better understanding the changes in different areas, we analyzed the changes per group, summarized in Table 1. As shown in Figure 14, the mean changes from patch A, patch B, patch C and patch D to “Ta6”, are −66.35, −31.08, −31.86 and −4.96 mm, respectively. The mean changes from patch A, patch B, patch C and patch D to “Ta7”, are −58.65, −30.07, −37.87 and −6.58 mm, respectively. The results indicate that the four patches moved towards “Ta6” and “Ta7”. However, the absolute values are quite different and show that patch A, B and C have a more obvious tendency towards “Ta6” and “Ta7” than patch D.

The mean change from patch A, patch B, patch C and patch D to “Ta3”, are 54.11 mm, −9.57 mm, 29.25 mm and 0.99 mm, respectively. “Ta3” is at the middle of the four patches along the X axis which lead to a different change compared to “Ta6” and “Ta7”: patch A and patch C moved away from the “Ta3” after the seismic testing while patch B moved towards “Ta3”. Patch D has a small change w.r.t. “Ta3” compared to the standard deviation of the change. Therefore we cannot draw a significant conclusion on its movement relative to “Ta3”. 

#### 3.4.2. Baseline Changes within a Patch

**Figure 15:**
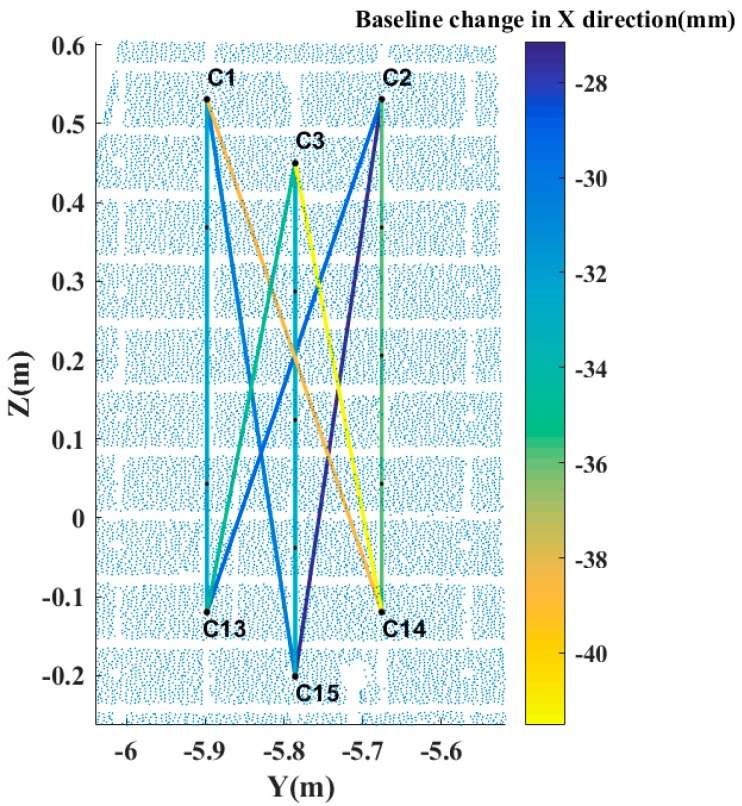
Baselines change in the X direction in patch C.

Obviously, the standard deviations of the mean change involving patch C are relatively large which indicate that damage happened within patch C. To validate this hypothesis, we select six virtual brick centre points, three in the top (C1, C2 and C3) and the other three in the bottom (C13, C14 and C15) of patch C. By linking virtual points in the top area to virtual points in the bottom area, 9 baselines for patch C are established and decomposed. Here the baseline direction is taken from the bottom area to the top area, which means the bottom area is taken as reference. The mean change of the baselines in patch C is −35.1, 0.0 and 0.6 mm in the X, Y and Z direction, respectively, in which the minus sign indicates that the change in the X direction is pointing into the moving building. Their standard deviations are 4.7, 0.9 and 0.1 mm, respectively. Because of the big change in the X direction, Figure 15 is plotted, which shows the baseline (in patch C) change in the X direction for a total of nine pairs of corresponding baselines between different brick centre points. It is concluded that damage happened to patch C in the X direction. 

#### 3.4.3. Baseline Changes between Patches

**Figure 16:**
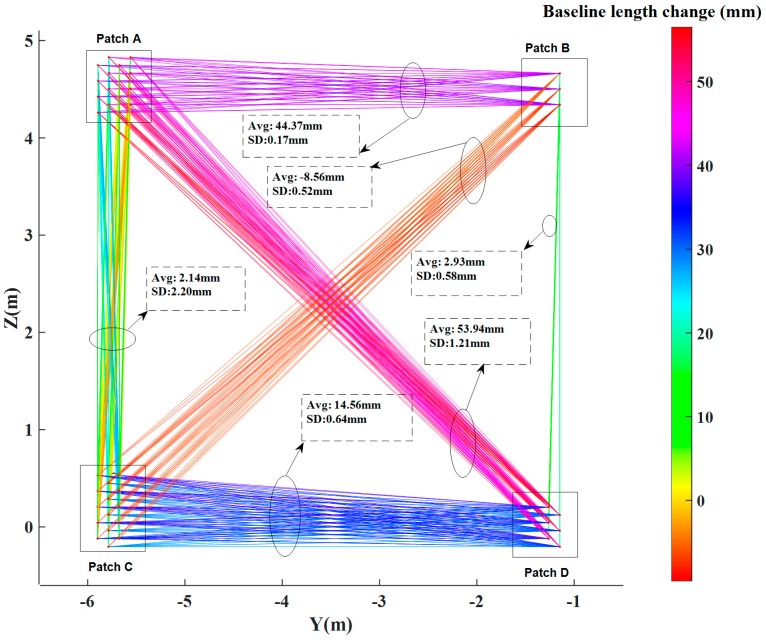
Baseline length changes between virtual brick centre points.

The variations in baselines connecting target points to virtual brick centre points indicate absolute change. While variation in baselines between different virtual points indicate relative change from one brick to the other. Figure 16 shows the length change for a total of 537 pairs of corresponding baselines between different virtual brick centre points, in which “Avg” represents the mean corresponding baselines length change and “SD” represents the standard deviation of corresponding baselines length changes. 

The change results are also summarized in Table 2. We selected the baselines from virtual brick centre points in patch B, C and D to virtual brick centre points in patch A, as examples to illustrate the change pattern. Patch D shows maximal change with regard to patch A. Its mean change is 53.94 mm. Patch C shows minimal change with regard to patch A. The mean change is 2.14 mm with a standard deviation of 2.20 mm. The mean change of patch B with regard to patch A is 44.37 mm with a much smaller standard deviation. The results indicate that patch B and D moved away from patch A after the seismic testing. 

#### 3.4.4. Displacement Vectors

**Figure 17:**
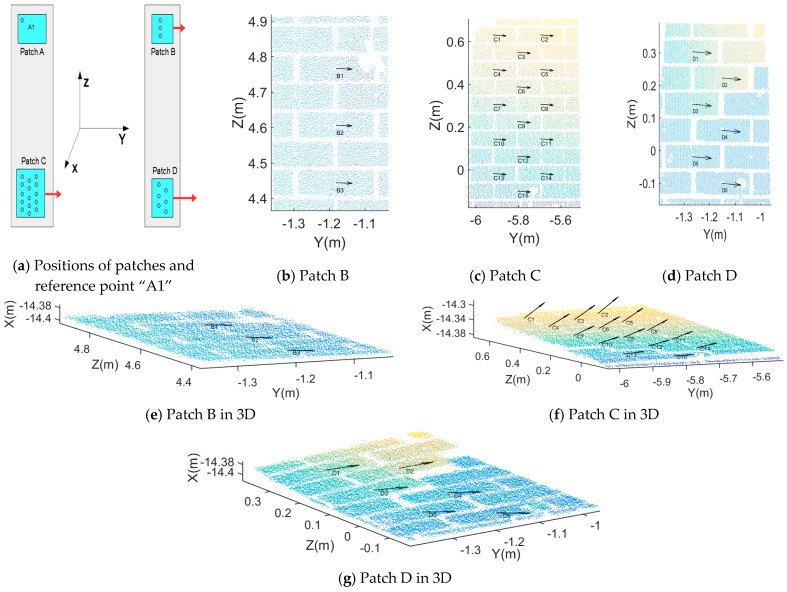
Displacement vectors. In the Figures (**b**–**d**) 2D vectors are given, indicating relative changes parallel to the affected wall. Full 3D vectors are given in Figures (**e**–**g**).

For easier interpretation of the detected changes, difference vectors between corresponding baselines are plotted relative to an arbitrary reference point “A1”. Figure 17a shows the position of point “A1” and the other three patches. The difference vectors from the perspective of X axis between corresponding baselines incident to “A1” are plotted in Figure 17b–d. However, these figures do not illustrate possible displacements in the X direction. Therefore, additional difference vectors in 3D between corresponding baselines incident to “A1” are plotted in Figure 17e–g.

By comparing the baselines projected to the X, Y and Z axes within the structural coordinate system, relative change information is obtained. Change is considered relative if it corresponds to change between different areas in the moving building, and therefore the change value here is absolute not relative. As regards the structural coordinate system, we have checked the difference in two epochs which is little enough to be ignored, namely this will not affect the results of our proposed methods. For easier to implement, we projected the baselines in two epochs respectively. Full change information is given in Table 3. Here we take the baselines connected to the virtual points in patch A as examples. The maximum change direction is along the Y axis in the structural coordinate system. The mean change values are −44.38, −58.97 and −73.33 mm for patch B, C, D, respectively, in which the minus sign denotes the direction in the opposite Y axis or left. Corresponding standard deviations are all well below 1 mm. Along the X axis, the mean change has a value of 2.82, −24.45 and 5.06 mm for patch B, C, D, respectively. The mean change value of −24.45 mm in patch C indicates that, compared to other patches, virtual brick centre points in patch C have a relatively large change along the X axis, which is pointing into the moving building. Compared to the other two directions, the mean change along the Z axis is less significant: Its values are −0.39, 2.45, 2.82 mm for patch B, C, D, respectively with in all three cases a standard deviation of ~0.8 mm. The above structural change results correspond well to the emerging cracks/damage shown in Figure 3. 

### 3.5. Comparison to Traditional Change Detection Methods

#### 3.5.1. Registration of Two Epochs

The control point registration is performed by the Leica Cyclone software. As introduced before, four plane targets were set. Control/tie points were additionally measured by the scanner, to reference the scans more accurately to a common coordinate system. However, the registration error of “Tag 5” was 0.010 m which was relatively high. Therefore, we have reason to assume that “Tag 5” was apparently moved by an external agent during the experiment. Considering that the scanner is always level, three targets are enough for registration. Thus, using the remaining three targets, the two point clouds are aligned which lead to a better result. The registration errors of the targets are shown in Table 4.

#### 3.5.2. Virtual Point Comparison

**Figure 18:**
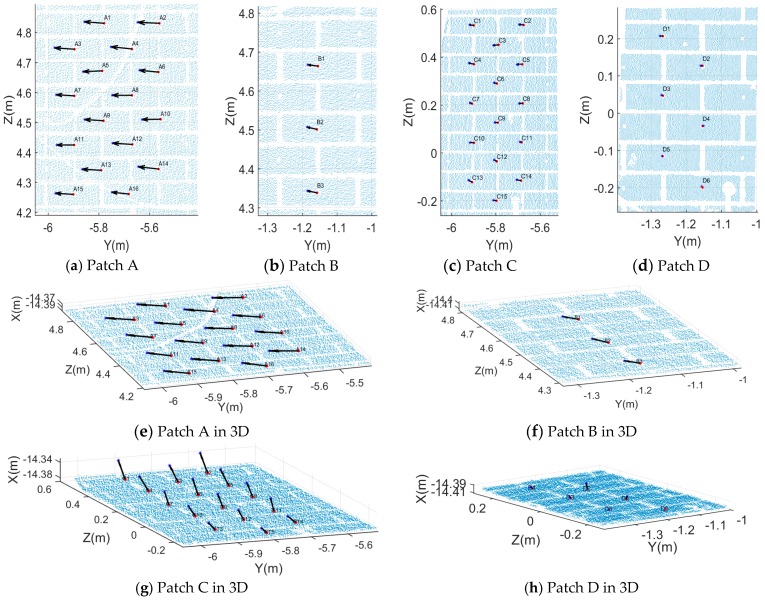
Direct virtual point comparison results.

After registration of the two epochs of data, all virtual brick centre points in each patch were extracted. We compared the 3D coordinates of the corresponding virtual points from two epochs and determined the change in X, Y and Z direction. Figure 18a–d show the change arrows for four patches projected on the YOZ plane. In patches C and D, the length of the change arrows is shorter than in patches A and B which indicates that the changes in Y and Z direction are small compared to patch C and patch D. In order to reveal changes in the X direction, change arrows for four patches are plotted in 3D in Figure 18e–h. From the above figures, we draw the conclusion that patch C moved in the X direction. Furthermore, there have a big difference of every virtual points in patch C which prove that damage happened in patch C not just shift movement.

In the top area (patch A and patch B), the maximum change direction is along the Y axis which is horizontal and parallel to the moving wall. While in the bottom area (patch C and patch D), the maximum change direction is in the X direction which is pointing into the moving wall. Along the Z direction (vertical direction), all four patches have a similar tendency: the change is marginal with a value of less than 5 mm. The change at the top area is slightly larger than at the bottom area. This means that after the seismic testing, no obvious vertical change of the moving wall occurred. In the Y direction, the top area (patch A and patch B) has a significantly greater change than the bottom area: the absolute displacement values of the four patches (A, B, C and D) in the Y direction are in [75.75 76.98] mm, [31.55 31.55] mm, [17.75 18.13] mm and [1.80 2.49] mm, respectively (In the symbol [], the left value is the minimum value in a patch while the right value is the maximum value in the same patch). Compared with ΔY, ΔZ, the pattern of ΔX has a big difference. The changes in patch A and patch B are smooth which indicate the patches shift as a whole, however, in patch C and patch D the changes vary prominently which indicates that apparent damage happened within patch C and patch D. The above virtual points comparison results provide information similar to the proposed baseline method and meanwhile verifies its effectiveness.

#### 3.5.3. Cloud-to-Cloud Distances

**Figure 19:**
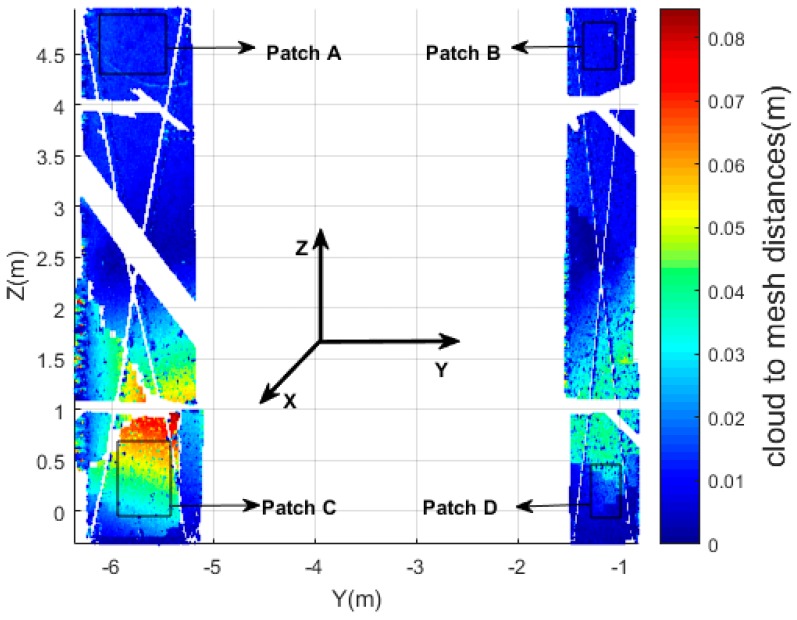
Scatter diagram of the cloud-to-cloud distances (entire wall).

First, we plot the cloud-to-cloud distances for the entire moving wall, see Figure 19. As the wall is approximately planar, a Least Squares best fitting plane is used while searching KNN points, where each leaf node contains a number of predefined target points. In this research, six nearby points were used. The maximum cloud-to-cloud distance is 0.0846 m in the bottom-left area. Actually, we can easily draw the conclusion from Figure 19 that just using cloud-to-cloud distances gives incomplete information on those areas that got internally damaged. Indeed, changes parallel to a wall are difficult to identify. Figure 19 heights change parallel to the wall.

**Figure 20:**
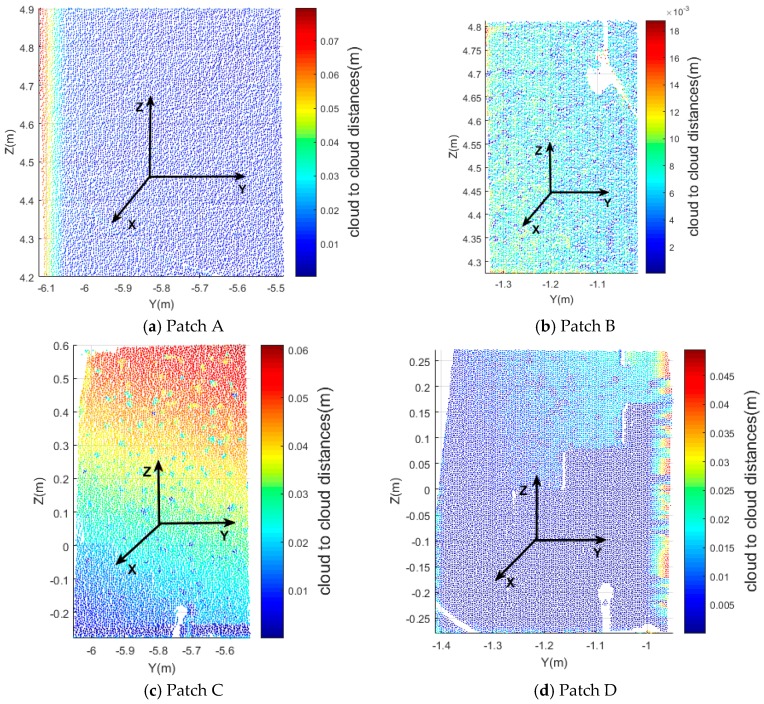
Scatter diagram of cloud-to-cloud distances.

Figure 20 shows the cloud-to-cloud distances for the four patches (A, B, C, D). As shown in Section 2.3.3, cloud-to-cloud distances are nearest neighbor distances not the distances between the corresponding points or features, therefore the change values are different from the baseline method and virtual point comparison. Please note that the cloud-to-cloud distances are absolute values, and therefore have no sign, in contrast to the change vectors obtained by the baseline method. The maximum cloud-to-cloud distances of patch A (top-left), B (top-right), C (bottom-left), and D (bottom-right) are about 0.076, 0.018, 0.061 and 0.050 m, respectively. In patch A, the distances are consistent from left to right. The biggest change is on the left side of the patch which shows the uniform move to the left, namely in the opposite Y direction. In patch B, there is a hole without points which is the results of an occlusion by a piece of wood. The cloud-to-cloud distance of patch B is small compared to the other patches. In patch C, the distances on the top are greater than on the bottom which indicates the damage that happened in this area is significant which corresponds to the results discussed in Section 3.4.2. In the middle of patch D, the distances are not consistent (an obvious fault exists). However, the absolute values of the distances are relatively low which indicates that only small damage happened in this area. 

#### 3.5.4. Comparative Analysis

From the above analysis it follows that the three methods give similar results in terms of maximum change, mean change and change tendency which directly indicates the feasibility of our proposed method. However, in our experiment, we could make use of available stable targets to obtain a good registration. If, for some practical reason, a scene does not have proper locations for targets, misalignments will affect the results of traditional methods while our proposed method will not suffer. In addition, the results clearly demonstrate some shortcomings of traditional change detection methods. Cloud-to-cloud distances are sensitive to the configuration of the compared point clouds. Insertion or removal of small objects, like the cables visible in Figure 12, will locally result in large cloud to cloud distances. In addition, cloud to cloud comparison is not suitable for revealing motion parallel to for example a wall. Comparing virtual points extracted from registered point clouds overcomes these issues, as virtual point comparison is fully 3D, while virtual point extraction implicitly ignores unwanted objects like the discussed cables. Still, comparing coordinates from different point clouds requires a reliable registration, which is not always achievable in practice. It should be noted though that also virtual point extraction introduces one or even two additional steps, the preferably automatic extraction of reconstructable points, and the identification of corresponding reconstructable points between epochs. The possibility to do so will strongly depend on the scene considered, but many scenes do have candidate virtual points in practice. It should be noted that the methods illustrated in this paper are largely automated. Only selecting the corresponding patches required manual work, but very precise selection was not required.

## 4. Conclusions and Recommendations

In this work we have proposed two new approaches to change detection in scenes sampled by laser scanning. Both of these approaches use so-called virtual points. For us, virtual points are 3D locations of features that are reconstructable from the available point cloud data. To enable comparison of virtual points, it is required that correspondences are established between the same virtual points in different scenes. One way of using virtual points is by directly comparing 3D coordinates between corresponding virtual points in different point clouds after registration. Advantage of this approach is that changes between well-established features are considered. Alternatively, baselines, i.e., lines connecting virtual points in one scene, are extracted and corresponding baselines from different epochs are compared to identify 3D change. This approach has the additional advantage that registration is no longer required.

For interpretation of the results in the latter case, we defined a so-called structural coordinate system. This coordinate system is used to decompose 3D change vectors into orthogonal components parallel and perpendicular to the object under consideration. In our case this object is the wall subject to change Definition and use of such coordinate system is essential to properly understand detected movement, which in general is a combination of rotation and displacement. As the baseline method identifies relative motion, the use of such structural coordinate system is also essential for the identification of absolute movement, as for this an external point of view is required outside the area that is subject to change. One could therefore argue that adding this structural coordinate system is a kind of back-door registration. Indeed, it does add additional information on the relative alignment of the scene in different epochs. Still, adding a structural coordinate system has less requirements and is less labor intensive then performing a fine registration. 

Both new methods were demonstrated on a masonry wall located at the TU Delft Stevin laboratory subject to seismic testing. The wall was scanned before and also after the seismic tests which caused obvious damage. The bricks constituting the masonry wall are clear, recognizable objects that are in addition of a suitable size for a structural damage assessment. Therefore, an algorithm was developed that automatically estimates the brick centre locations from the laser scan intensity data. The intensity response allowed us to separate mortar points from brick points using k-means clustering. Next, brick outlines and brick centre locations are estimated from projected point densities of the mortar points. Baselines connecting brick centres and targets in different scans were established, decomposed and analyzed. 

The results clearly indicate how different parts of the damaged wall move in 3D with respect to each other. By identifying a larger spread of differences in lengths of baselines all connected to one part of the wall, very local damage, almost at brick level, could be identified in a next, more local analysis. An obvious disadvantage of traditional cloud to cloud based change detection is that changes parallel to a surface are difficult to detect. In that sense cloud to cloud based change detection is a 2.5 D method while both the virtual point method and the baseline method are truly 3D as it reports 3D change vectors as a result. Designing a suitable structural coordinate system with axes parallel and perpendicular to the wall under study in this case helped the interpretation of the results.

The two proposed methods are general in the sense that they could be applied to any situation where repeated scan data is available, provided that it is possible in the scene under study to identify the corresponding features. We believe that identification of features is indeed possible in many man-made objects, which makes also the proposed method widely applicable, e.g., [46,47]. It should also be possible to extract features in e.g., trees, outcrops and terrain covered by boulders, so the method should also work for geomorphological application. In addition, we have to demonstrate that for different scenarios it is indeed possible to establish a suitable structural coordinate system, which may be difficult in the open area with ragged topography. Still, to make the method powerful would require to have a supporting method that automatically extracts and identify suitable features in the given application, like in this paper the brick centres extraction algorithm. What should also be studied in future are (i) methods to estimate the quality of the feature points, which could be; (ii) propagated to the quality of the extracted baselined, to be finally used to (iii), test whether a change in baseline is significant [19].

## Figures and Tables

**Table 1:** Baseline change between patches and targets.

Target	Patch	Maximum Change (mm)	Minimum Change (mm)	Mean Change (mm)	Standard Deviation (mm)
Ta3	A	57.86	50.70	54.11	2.05
	B	−10.09	−8.98	−9.57	0.56
	C	41.36	17.04	29.25	7.32
	D	−4.81	−1.10	0.99	2.92
Ta6	A	−67.79	−64.14	−66.35	1.07
	B	−31.28	−30.95	−31.08	0.17
	C	−41.32	−21.02	−31.86	6.14
	D	−7.42	−3.32	−4.96	1.64
Ta7	A	−59.88	−56.48	−58.65	1.00
	B	−30.77	−30.46	−30.57	0.17
	C	−51.81	−22.18	−37.87	9.10
	D	−10.54	−3.79	−6.58	2.72

**Table 2:** Baseline change between patches.

Patch	Patch	Maximum Change (mm)	Minimum Change (mm)	Mean Change (mm)	Standard Deviation (mm)
A	B	44.63	44.07	44.37	0.17
	C	6.93	0.06	2.14	2.20
	D	56.50	50.87	53.94	1.21
B	C	−9.60	−7.66	−8.56	0.52
	D	3.61	2.05	2.93	0.58
C	D	15.49	13.33	14.56	0.64

**Table 3:** Displacement of baselines connected to patch A.

Patch	Patch	Axis	Maximum Change (mm)	Minimum Change (mm)	Mean Change (mm)	Standard Deviation (mm)
A	B	X	4.40	0.56	2.82	1.00
		Y	−44.60	−44.21	−44.38	0.16
		Z	1.61	0.01	−0.39	0.81
	C	X	−46.36	−1.33	−24.45	12.60
		Y	−60.00	−58.23	−58.97	0.61
		Z	4.77	1.38	2.45	0.83
	D	X	12.11	0.07	5.06	4.98
		Y	−73.71	−73.00	−73.33	0.23
		Z	5.24	1.67	2.82	0.87

**Table 4:** Automatic registration error by Leica Cyclone software.

Target Name	Weight	Error (m)	Error Vector (m)
Ta3	1	0.001	(0.001,0.000,0.000)
Ta6	1	0.000	(−0.001,0.000,0.000)
Ta7	1	0.001	(0.000,0.000,0.000)
